# A New Curve of Critical Leaf Potassium Concentration Based on the Maximum Root Dry Matter for Diagnosing Potassium Nutritional Status of Sweet Potato

**DOI:** 10.3389/fpls.2021.714279

**Published:** 2021-09-30

**Authors:** Zunfu Lv, Guoquan Lu

**Affiliations:** The Key Laboratory for Quality Improvement of Agricultural Products of Zhejiang Province, College of Advanced Agricultural Sciences, Zhejiang A&F University, Hangzhou, China

**Keywords:** sweet potato, leaf, critical potassium concentration, K nutrition index (KNI), root

## Abstract

Critical leaf nutrient concentrations have often been used to diagnose the nutritional status of crops. Determining critical leaf potassium (K) concentrations for the maximum root dry matter (RDM) will provide a reliable means of linking leaf K nutrient concentrations to the yield of sweet potato. Three field experiments, using varying K application rates (0–300 kg K ha^−1^) and two sweet potato cultivars, were performed in the Zhejiang Province of China. A new critical leaf K curve (K_leaf_) based on the maximum RDM was determined to assess K nutrition in sweet potato and described by the equation ${\text{K}}_{\text{leaf}}=4.55\times \text{RD}{\text{M}}_{\max}^{-0.075}$. A critical root K curve (K_root_) based on the maximum RDM was also determined to assess K nutrition and described by the equation ${\text{K}}_{\text{root}}=2.36\times \text{RD}{\text{M}}_{\max}^{-0.087}$. The K nutrition index (KNI) was constructed to identify the situations of K-limiting and non-K-limiting treatments. The leaf KNI (KNI_leaf_) ranged from 0.56 to 1.17, and the root K KNI (KNI_root_) ranged from 0.52 to 1.35 during the growth period of sweet potato. The results showed that the critical leaf K concentration curve can be used as an accurate leaf K status diagnostic tool at critical growth stages that connected leaf nutrient concentration and sweet potato tuber yield. This K curve will contribute to K management of sweet potato during its growth period in China.

## Introduction

Sweet potato (*Ipomoea batatas* L.) is an essential food crop, and based on its cultivation area and yield, it is ranked as the fourth major food crop after rice, wheat, and corn in China (Ma et al., 2012). China cultivates sweet potato on a large scale, and the annual average total yield and planting area are ranked first in the world (Tang et al., 2018). Sweet potato is a typical potassium (K)-favoring crop. The demand ratio of nitrogen (N), phosphorus (P), and K is ~2:1:4 (Wang et al., 2015). Unlike N and P nutrients, K does not become a part of the chemical structure of the plant. Sweet potato is much more vulnerable to K-deficiency than to N- and P-deficiency (Tang et al., 2018). K plays many regulatory roles in plant nutrition, i.e., it promotes root growth, activates enzymes, improves photosynthetic capacity, transports sugar and starch, and causes earlier tuber initiation (Liu et al., 2013). Since K application plays a significant role in the distribution and accumulation of dry matter in tuber roots (Shi et al., 2002), it promotes the accumulation of K in sweet potato (Li et al., 2015) and increases tuber root yield (Ning et al., 2012; Wang et al., 2017a). However, excessive K application would lead to luxury absorption of K in sweet potato and a reduction in the K utilization rate (Foloni et al., 2013).

The concept of critical nutrient concentration was first proposed in 1984 (Lemaire and Salette, 1984) on N, and it was stated that a minimum N concentration is required for maximal growth. Models have been constructed to simulate critical N concentration dilution curves for rice (Ata-Ul-Karim et al., 2017), corn (Zhao et al., 2017), wheat (Yao et al., 2014), and other crops. However, the critical K concentration curve was rarely studied. The critical K concentration is defined as the minimum K concentration required for maximal growth. Some studies found that the K concentration in sweet potato decreases with an increase in biomass during the growth and development of sweet potato (Qi et al., 2016). In addition, some studies reported that the potato seedling dry weight and yield no longer increased when the K concentration exceeded a certain level (Trehan and Claassen, 1998; Karam et al., 2009). All these studies indicated the likely presence of a critical K concentration curve for crops. Recent studies by Adiele et al. (2021) reported, for the first time, the critical K dilution curves for cassava.

Leaf nutrient concentration is usually used to diagnose nutrient deficiency in crops. O'Sullivan et al. (1997) used leaf nutrient concentrations to diagnose nutrient disorders of sweet potato. However, these diagnostic criteria were not available for mature storage root of sweet potato. Ramakrishna et al. (2009) developed the Diagnosis and Recommendation Integrated System to provide a means of connecting leaf nutrient concentrations to crop yield, but it was based on empirical statistical methods. Ze and Dong (1991) showed that leaf nutrition excess or deficiency could determine the root yield of sweet potato, but it did not show the critical leaf value. The leaf nutrient concentration is, therefore, optimal at the highest root dry matter (RDM) values. Therefore, a critical K concentration in sweet potato leaves can be used to diagnose K nutrition in its storage roots.

Source-sink balance is an ideal state for sweet potato growth. The root/shoot (R/S) ratio is the best index to reflect the balance between source and sink (Ning et al., 2015). K application can promote the translocation of photosynthates to storage roots (Liu et al., 2013). The appropriate leaf K concentration is the key to coordinate the source-sink balance. K and other nutrients are the internal driving factors of sweet potato growth, and the R/S ratio is the external performance. The critical K concentration and the optimal R/S ratio represent the best physiological and growth state of sweet potato, respectively. The change of R/S ratio of sweet potato under different K concentrations is helpful to clarify the effect of K concentration on R/S ratio.

The objectives of this study were to (i) determine the critical leaf K concentrations for sweet potato for maximum RDM, (ii) construct a K nutrition index (KNI) for assessing K nutritional status, and (iii) reveal the relationship between shoots and roots under different K fertilization levels.

## Materials and Methods

### Experimental Design

Three field experiments, using varying K application rates and two sweet potato cultivars (Xinxiang and Shang 19), were conducted at Lin'an (30°15′N, 119°43′E) and Yuhang (30°41′N, 120°29′E) in the Zhejiang Province of China. The basic soil data and that of the three field experiments are shown in Table 1. The soil was clay loam, classified as Ultisol soil. The annual average temperature is 16.4°C. The annual average precipitation is 1,628.6 mm, and precipitation occurs mostly between April and September. The minimum and maximum temperatures recorded in the sampling date are shown in Table 1. The plot size was 6 × 4 m, and plots were arranged in a randomized block design with three replicates. The planting density was 5.0 plants m^−2^ with a distance of 1 m between plants and 0.2 m between rows. As shown in Table 1, organic matter, total N, available K, and available *P* values were determined per site and year. Five different K application rates consisting of 0 (K_0_), 75 (K_1_), 150 (K_2_), 225 (K_3_), and 300 kg ha^−1^ (K_4_) were used. Notably, 50% K-fertilizer was applied as base fertilizer, and 50% K-fertilizer was applied on day 30 after planting (50%). N (50 kg ha^−1^) and P (120 kg ha^−1^) fertilizers were applied as base fertilizers in all plots. N (50 kg ha^−1^) fertilizers were dressed on day 30 after planting. Planting occurred on May 4, 2017, April 30, 2018, and May 1, 2019. The data used to construct critical K concentration curves were derived from Experiments 2 and 3. The data were used to validate the critical K concentration curves from Experiment 1.

**Table 1:** Nutrient content data for the three field experiments.

**Experiment no**.	**Site**	**Cultivar**	**K rate** **(kg ha**^**−1**^**)**	**Sampling date** **(min-max temperature, **^**°**^**C)**	**Organic matter** **(g kg**^**−1**^**)**	**Total N** **(g kg**^**−1**^**)**	**Available P** **(mg kg**^**−1**^**)**	**Available K** **(mg kg**^**−1**^**)**
Experiment 12017	Yuhang (30°41′N, 120°29′E)	XinxiangShang 19	K_0_ (0) K_1_ (75) K_2_ (150) K_3_ (225) K_4_ (300)	30 (20-31), 44 (20-28), 59 (26-34), 76 (29-38), 92 (28-38), 104 (26-34)	14.1	0.80	40	71
Experiment 22018	Lin'an (30°15′N, 119°43′E)	XinxiangShang 19	K_0_ (0) K_1_ (75) K_2_ (150) K_3_ (225) K_4_ (300)	39 (22-27), 52 (21-30), 67 (25-30), 81 (27-36), 101 (27-36), 123 (26-35)	18.7	0.94	37	80
Experiment 32019	Lin'an (30°15′N, 119°43′E)	XinxiangShang 19	K_0_ (0) K_1_ (75) K_2_ (150) K_3_ (225) K_4_ (300)	44 (18-27), 55 (23-25), 63 (23-28), 78 (26-34), 99 (28-38), 120 (24-32)	16.3	0.81	42	74

*N, nitrogen; P, phosphorus; K, potassium*.

### Sampling and Measurements

Sweet potato samples were collected from the experimental plots during six growth dates, and five sweet potato plants were randomly selected from each plot. The leaf, stem, and root samples of sweet potato were dried to constant mass at 80°C, and the dry biomass was determined. Roots and corresponding aboveground biomass were used to calculate the R/S ratio. The separated organs of the dried sweet potato plants were ground to powder to pass through a 1-mm sieve. The powder (0.200 ± 0.020 g) was weighted out to measure K content. The leaf K content (LKC) and the root K content were determined using atomic absorption spectroscopy (Thermo Scientific iCE™ 3300, Germany).

### Model Construction

####  Critical Leaf K Concentration Based on the Maximum RDM

The critical leaf K concentration was constructed based on the critical data points at which RDM was neither limited nor enhanced by K. A critical data point was determined by each sampling data that include K-limiting treatments and non-K-limiting treatments. With increasing K application, both the storage root weight and leaf K concentration of sweet potato increased significantly (*P* < 0.05). With the continuous increase of K application, there were no significant changes in the storage root weight (*P* < 0.05), but the leaf K concentration increases continuously with increasing K application (*P* < 0.05). Therefore, the K treatments for each sampling were divided into two groups: (i) K-limiting treatment was defined as a treatment in which additional K supply led to a significant response in the storage root weight and leaf K concentration and (ii) non-K-limiting treatment was defined as a treatment in which additional K supply did not lead to a significant increase in the storage root weight but resulted in a significant increase in the leaf K concentration. The RDM values in different K treatments were compared using ANOVA (SPSS Statistics 19.0 software, IBM Corp., Armonk, NY, USA) at a probability level of 5%.

The critical leaf K concentration based on the maximum RDM is defined as the minimum leaf K concentration required for maximal root growth. A critical leaf K concentration model for sweet potato roots was constructed according to the method of the critical N concentration model (Justes et al., 1994). First, the RDM and the leaf K concentrations were used to identify whether root growth was restricted by K nutrition. A regression line was used to fit data from the K-limiting treatments. A vertical line was calculated with the data from the non-K-limiting treatments as the average of the maximum RDM. The critical leaf K concentration point was the intersection point of the oblique and vertical lines. The following equation describes the critical leaf K concentration curve (K_leaf_) based on the maximum RDM:


(1) $$\begin{array}{l} {\text{K}}_{\text{leaf}}={\text{a}}_{1}\times \text{RD}{\text{M}}_{\max}^{-b1} \end{array}$$

where a_1_ and b_1_ are the empirical coefficients.

#### Critical Root K Concentration Based on the Maximum RDM

The critical root K concentration is defined as the minimum root K concentration required for maximal root growth. A critical root K concentration model for sweet potato was constructed according to the method of the critical root N concentration model (Lv et al., 2020). The following equation describes the critical root K concentration curve (K_root_) based on the maximum RDM:


(2) $$\begin{array}{l} {\text{K}}_{\text{root}}={\text{a}}_{2}\times \text{RD}{\text{M}}_{\max}^{-b2} \end{array}$$

where a_2_ and b_2_ are the empirical coefficients.

#### The KNI

The KNI is an indicator used to determine crop K status. The KNI_leaf_ at each sampling date was determined according to the actual leaf K concentration and the critical leaf K concentration as follows:


(3) $$\begin{array}{l} {\text{KNI}}_{\text{leaf}}={\text{K}}_{\text{l}}/{\text{K}}_{\text{leaf}} \end{array}$$

The KNI_root_ at each sampling date was determined according to the actual root K concentration and the critical root K concentration as follows:


(4) $$\begin{array}{l} {\text{KNI}}_{\text{root}}={\text{K}}_{\text{r}}/{\text{K}}_{\text{root}} \end{array}$$

where K_l_ and K_r_ are the measured leaf and root K concentrations, respectively. Thus, for KNI = 1, K nutrition is considered optimal for root growth; for KNI <1, K nutrition is considered limited for root growth, and for KNI > 1, K nutrition is considered excessive for root growth plants contain excess K.

The R/S ratio reflects the relationship between the roots and the shoots. The allometric growth of root and shoot can be expressed as the following equation: R = a × S^b^.

## Results

### RDM According to K Application Rate

**Figure 1:**
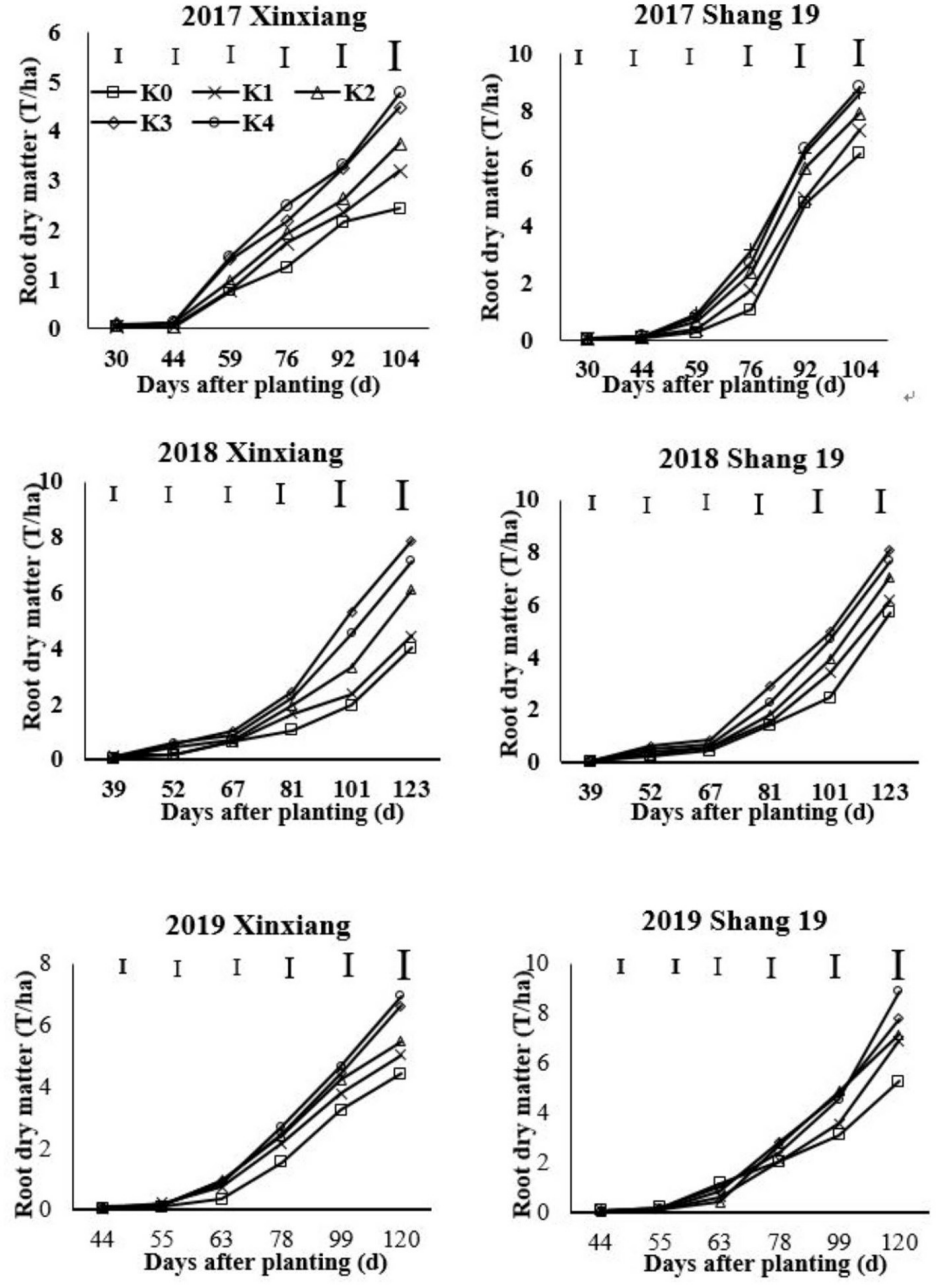
Changes in root dry matter (RDM) for two sweet potato cultivars with time under different potassium (K) fertilization levels.

The RDM of sweet potato gradually increased during the growth period of sweet potato (Figure 1; Table 2). The growth rate increased significantly after 2 months of planting. The RDM showed a continuously increasing trend from K_0_ to K_3_ treatments, but there were no significant differences among K_3_ and K_4_ treatments. Therefore, RDM increased initially and then stabilized as the K application rate increased for two sweet potato cultivars. RDM differed significantly among K_0_, K_1_, and K_2_ treatments. The RDM in K_3_ and K_4_ treatments with high K application rates was significantly higher than that in the three treatments with low K application rates. The RDM was consistent with the following inequality under different K application treatments: K_0_ < K_1_ < K_2_ < K_3_ = K_4_.

**Table 2:** Root dry matter for sweet potato grown under different potassium (K) application rates in three field experiments.

**Cultivars**	**DAP**	**Root dry matter (T/ha)**					**Cultivars**	**DAP**	**Root dry matter (T/ha)**				
**Year**		**K_**0**_**	**Year**	**K_**2**_**	**K_**3**_**	**K_**4**_**	**Year**		**K_**0**_**	**K_**1**_**	**K_**2**_**	**K_**3**_**	**K_**4**_**
Xinxiang	30	0.03d	0.04c	0.07b	0.09a	0.05c	Shang 19	30	0.05a	0.05a	0.02b	0.05a	0.02b
2017	44	0.03c	0.06b	0.13a	0.12a	0.12a	2017	44	0.08c	0.11b	0.11b	0.14a	0.14a
	59	0.76c	0.79c	0.97b	1.42a	1.46a		59	0.27c	0.38c	0.63b	0.92a	0.83ab
	76	1.24e	1.74d	1.93c	2.17b	2.50a		76	1.06e	1.73d	2.35c	3.13a	2.70b
	92	2.15d	2.36c	2.64b	3.25a	3.31a		92	4.77c	4.95c	6.01b	6.55a	6.70a
	104	2.43e	3.20d	3.75c	4.48b	4.77a		104	6.53c	7.34b	7.93b	8.63a	8.85a
Xinxiang	39	0.05c	0.16a	0.07c	0.05c	0.10b	Shang 19	39	0.02c	0.05bc	0.04c	0.06a	0.06a
2018	52	0.17c	0.19c	0.43b	0.55a	0.60a	2018	52	0.23d	0.25d	0.36c	0.60a	0.48b
	67	0.64c	0.64c	0.74c	1.03a	0.88b		67	0.44d	0.55c	0.56c	0.82a	0.67b
	81	1.05e	1.64d	1.96c	2.44a	2.24b		81	1.42d	1.51d	1.82c	2.90a	2.28b
	101	1.95e	2.37d	3.30c	5.31a	4.53b		101	2.45e	3.40d	3.92c	4.95a	4.66b
	123	4.02e	4.45d	6.11c	7.88a	7.15b		123	5.70e	6.15d	7.05c	8.10a	7.65b
Xinxiang	44	0.03d	0.05c	0.08b	0.10a	0.05c	Shang 19	44	0.03d	0.04c	0.06b	0.08a	0.04c
2019	55	0.09c	0.20a	0.14b	0.13b	0.13b	2019	55	0.07c	0.16a	0.11b	0.10b	0.10b
	63	0.34d	0.74c	0.94a	0.92a	0.85b		63	0.27d	0.57c	0.73a	0.71ab	0.66b
	78	1.55d	2.14c	2.41b	2.48b	2.68a		78	1.20d	1.67c	1.87b	1.93b	2.09a
	99	3.22e	3.78d	4.21c	4.43b	4.65a		99	2.51e	2.94d	3.28b	2.82c	3.62a
	120	4.41e	5.02d	5.47c	6.62b	6.95a		120	2.86e	3.91d	4.26c	5.15b	5.40a

*Different letters behind the numbers indicate the significance at P ≤ 0.05.*

*DAP, days after planting*.

### Leaves and Roots K Concentration Under Different K-Fertilizer Level

**Figure 2:**
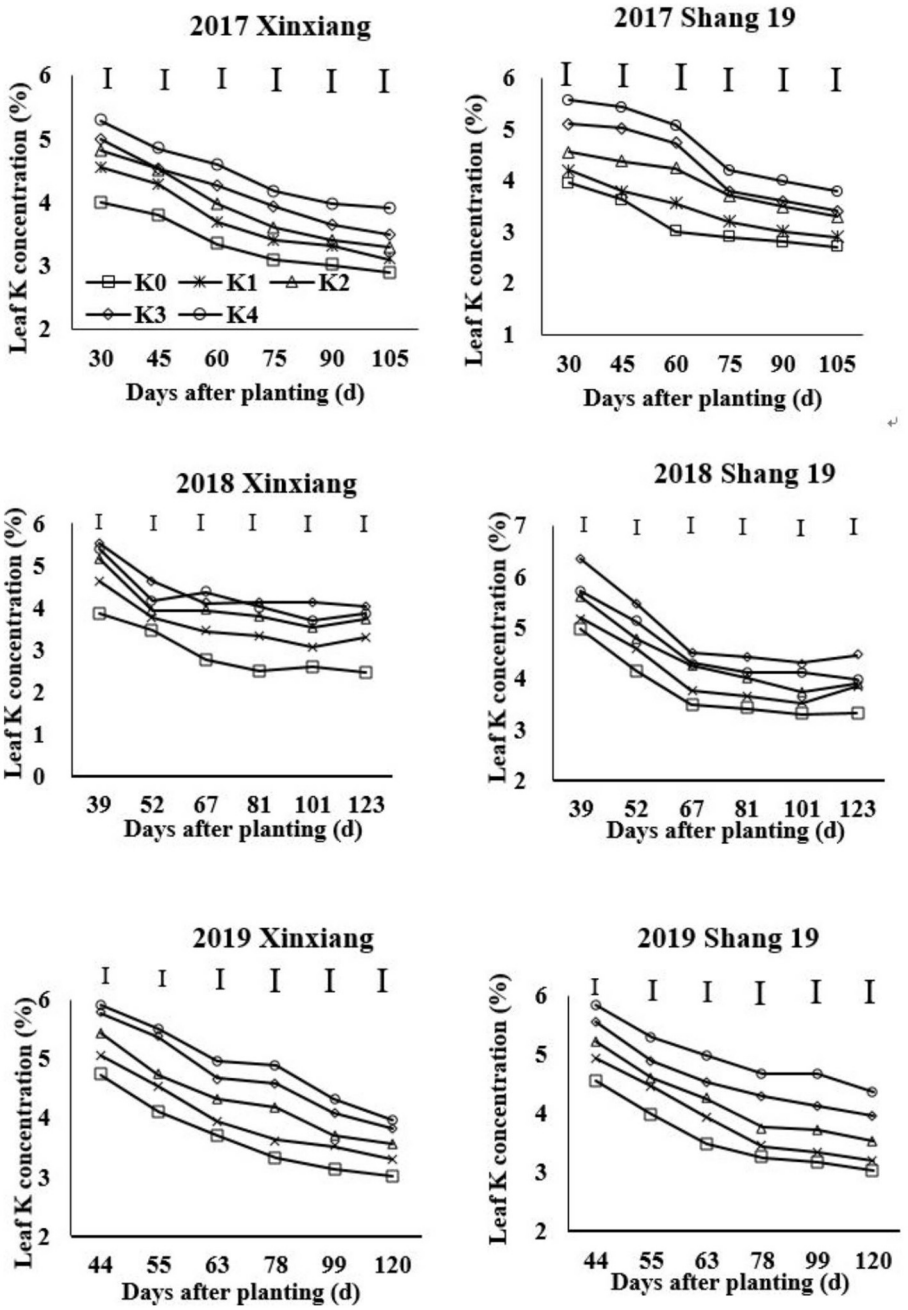
Changes in K concentration in sweet potato leaves with time under different K fertilization levels.

The leaf K concentration declined gradually during the growth period of sweet potato (Figure 2; Table 3). The K concentration in sweet potato leaves increased as the K application rate increased. Leaf K concentration showed a slightly decreasing trend during days 67–123 in 2018 and days 63–120 in 2019 (Figure 2). Leaf K concentration ranged from 2.9 to 5.29% for the Xinxiang cultivar and 2.7 to 5.57% for Shang 19 in 2017, from 2.47 to 5.39% for the Xinxiang cultivar and 2.33 to 6.36% for Shang 19 in 2018, and from 3.01 to 5.91% and 3.03 to 5.85%, respectively, in 2019 (Figure 2).

**Table 3:** Leaf K concentration for sweet potato grown under different K application rates in three field experiments.

**Cultivars**	**DAP**	**Leaf K concentration (%)**					**Cultivars**	**DAP**	**Leaf K concentration (%)**				
**Year**		**K_**0**_**	**K_**1**_**	**K_**2**_**	**K_**3**_**	**K_**4**_**	**Year**		**K_**0**_**	**K_**1**_**	**K_**2**_**	**K_**3**_**	**K_**4**_**
Xinxiang	30	4.01e	4.56d	4.81c	5.00a	5.29a	Shang 19	30	3.96e	4.20d	4.57c	5.12b	5.57a
2017	45	3.81d	4.30c	4.52b	4.53b	4.85a	2017	45	3.63e	3.79d	4.38c	5.03b	5.44a
	60	3.35e	3.68d	3.99c	4.28b	4.61a		60	3.00e	3.55d	4.25c	4.73b	5.07a
	75	3.09e	3.41d	3.60c	3.94b	4.18a		75	2.90d	3.20c	3.70b	3.80b	4.20a
	90	3.01d	3.32c	3.40c	3.64b	3.99a		90	2.80d	3.00c	3.50b	3.60b	4.00a
	105	2.90e	3.10d	3.30c	3.50b	3.91a		105	2.70d	2.90c	3.30b	3.40b	3.80a
Xinxiang	39	3.86e	4.62d	5.16c	5.54a	5.39b	Shang 19	39	4.98d	5.19c	5.62b	6.36a	5.73b
2018	52	3.47e	3.76d	3.93c	4.62a	4.16b	2018	52	4.14e	4.58d	4.78c	5.46a	5.13b
	67	2.76e	3.44d	3.94c	4.11b	4.38a		67	3.48d	3.78c	4.25b	4.50a	4.31b
	81	2.50e	3.33d	3.79c	4.13a	4.01a		81	3.41d	3.64c	4.01b	4.42a	4.12b
	101	2.60d	3.05c	3.52b	4.14a	3.70b		101	3.30c	3.53b	3.73b	4.31a	4.11a
	123	2.47d	3.29c	3.72b	4.03a	3.86b		123	3.33c	3.83b	3.90b	4.47a	3.99b
Xinxiang	44	4.74d	5.07c	5.45b	5.78a	5.91a	Shang 19	44	4.56e	4.94d	5.22c	5.56b	5.85a
2019	55	4.11d	4.54c	4.74c	5.38b	5.52a	2019	55	3.98d	4.46c	4.61c	4.89b	5.29a
	63	3.7e	3.95d	4.33c	4.67b	4.97a		63	3.48e	3.94d	4.26c	4.54b	4.99a
	78	3.32e	3.63d	4.18c	4.59b	4.89a		78	3.25d	3.44d	3.76c	4.29b	4.68a
	99	3.13e	3.52d	3.7c	4.08b	4.32a		99	3.18d	3.33d	3.73c	4.14b	4.67a
	120	3.01d	3.3c	3.57b	3.83a	3.96a		120	3.03d	3.2d	3.54c	3.97b	4.37a

*Different letters behind the numbers indicate the significance at P ≤ 0.05.*

*DAP, days after planting*.

**Figure 3:**
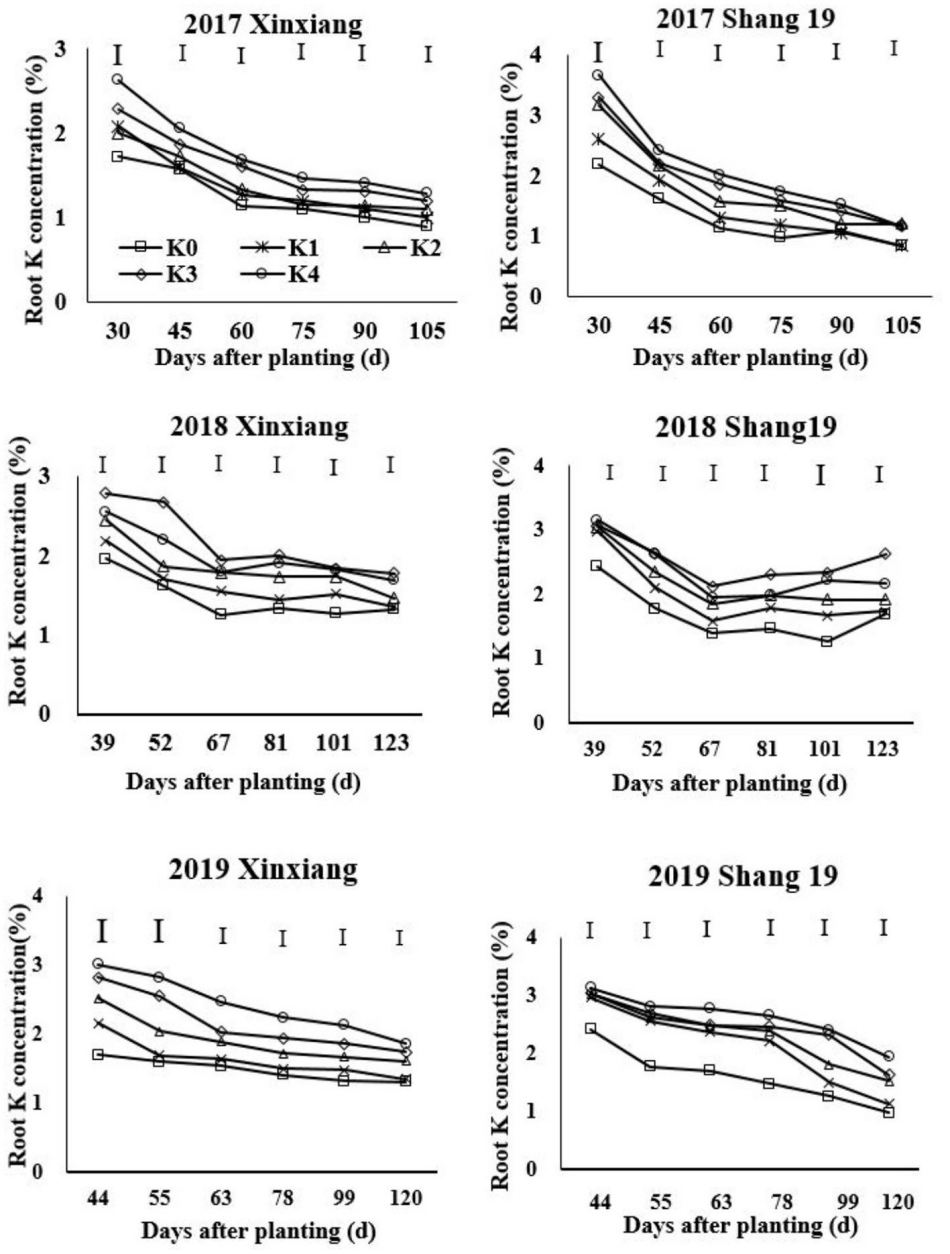
Changes in K concentration in sweet potato roots with time under different K fertilization levels.

The root K concentration declined gradually during the growth period of sweet potato (Figure 3; Table 4). The K concentration in sweet potato roots increased with the increasing K application rate (Figure 3). The root K concentration exhibited a significant decreasing trend during days 39–67 in 2018 and during days 44–63 in 2019, but it exhibited a slightly decreasing trend during days 67–123 in 2018 and days 63–120 in 2019 (Figure 3). Root K concentration ranged from 0.9 to 2.63% for the Xinxiang cultivar and 0.84 to 3.67% for Shang 19 during 2017, from 1.36 to 2.79% for the Xinxiang cultivar and 1.26 to 3.16% for Shang 19 during 2018, and from 1.35 to 3.02% and 1.33 to 3.13%, respectively, in 2019 (Figure 3).

**Table 4:** Root K concentration for sweet potato grown under different K application rates in three field experiments.

**Cultivars**	**DAP**	**Root K concentration (%)**					**Cultivars**	**DAP**	**Root K concentration (%)**				
**Year**		**K_**0**_**	**K_**1**_**	**K_**2**_**	**K_**3**_**	**K_**4**_**	**Year**		**K_**0**_**	**K_**1**_**	**K_**2**_**	**K_**3**_**	**K_**4**_**
Xinxiang	30	1.73d	2.08c	2.00c	2.29b	2.63a	Shang 19	30	2.19e	2.6d	3.18c	3.3b	3.67a
2017	45	1.58c	1.60bc	1.73b	1.87b	2.06a	2017	45	1.61d	1.92c	2.17b	2.20b	2.42a
	60	1.14c	1.27bc	1.34b	1.61a	1.69a		60	1.14e	1.30d	1.57c	1.85b	2.01a
	75	1.10c	1.20c	1.16c	1.33b	1.47a		75	0.97e	1.18d	1.5c	1.59b	1.74a
	90	1.00d	1.10c	1.14c	1.32b	1.42a		90	1.08d	1.05d	1.2c	1.41b	1.52a
	105	0.90e	1.00d	1.10c	1.20b	1.29a		105	0.83b	0.84b	1.2a	1.15a	1.16a
Xinxiang	39	1.96d	2.18c	2.45b	2.79a	2.55b	Shang 19	39	2.44c	2.99b	3.05b	3.07b	3.16a
2018	52	1.62d	1.70d	1.86c	2.67a	2.20b	2018	52	1.78d	2.10c	2.35b	2.64a	2.63a
	67	1.26d	1.55c	1.78b	1.94a	1.79b		67	1.40d	1.58c	1.86b	2.12a	1.96b
	81	1.33d	1.44c	1.73b	2.00a	1.91a		81	1.47d	1.80c	1.98b	2.31a	1.99b
	101	1.27d	1.52c	1.73b	1.84a	1.82a		101	1.27d	1.67c	1.92b	2.34a	2.23a
	123	1.32c	1.36c	1.46b	1.77a	1.69a		123	1.70d	1.74d	1.92c	2.63a	2.17b
Xinxiang	44	1.70d	2.16c	2.53b	2.83a	3.02a	Shang 19	44	2.42c	2.96b	3.02ab	3.04ab	3.13a
2019	55	1.60d	1.69d	2.04c	2.55b	2.83a	2019	55	1.77d	2.56c	2.62bc	2.69b	2.81a
	63	1.55e	1.64d	1.89c	2.03b	2.48a		63	1.70d	2.36c	2.48b	2.49b	2.77a
	78	1.42d	1.50d	1.72c	1.95b	2.25a		78	1.48d	2.22c	2.38b	2.46b	2.66a
	99	1.32e	1.48d	1.67c	1.87b	2.14a		99	1.26d	1.50c	1.81b	2.32a	2.41a
	120	1.31d	1.35d	1.61c	1.74b	1.86a		120	0.98e	1.13d	1.52c	1.63b	1.94a

*Different letters behind the numbers indicate the significance at P ≤ 0.05.*

*DAP, days after planting*.

### Critical Leaf K Concentration Based on RDM

**Figure 4:**
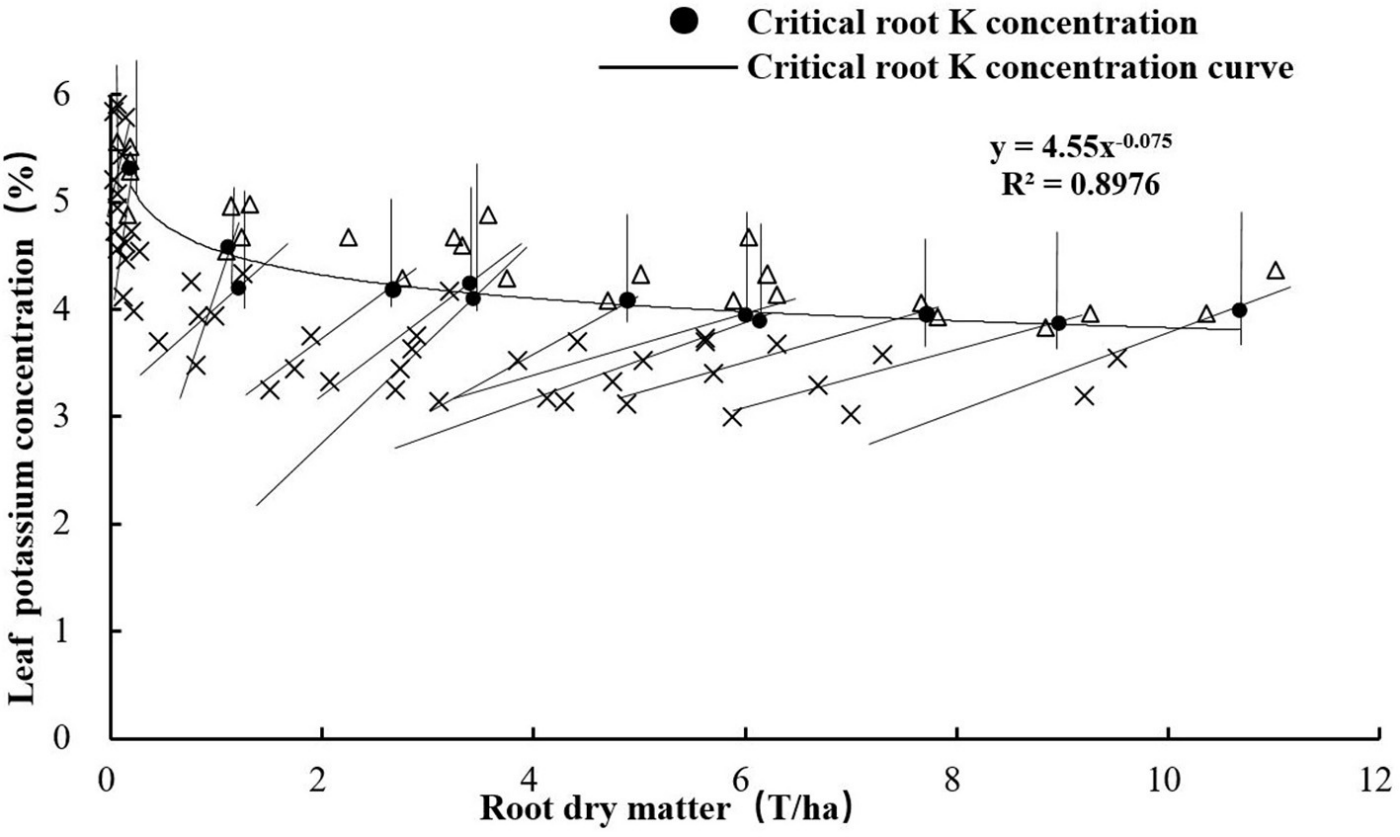
Critical leaf K concentration curve based on RDM.

According to the method described in the “Model construction” section, the critical leaf K concentration point was the intersection point of the oblique and vertical lines (Figure 4). There was a decreasing trend of K_leaf_ with increasing RDM of sweet potato. The regression trend line could be fitted by the following power equation:


(5) $$\begin{array}{l} {\text{K}}_{\text{leaf}}=4.55\text{ RD}{\text{M}}^{-0.075} \end{array}$$

### Critical Root K Concentration Based on RDM

**Figure 5:**
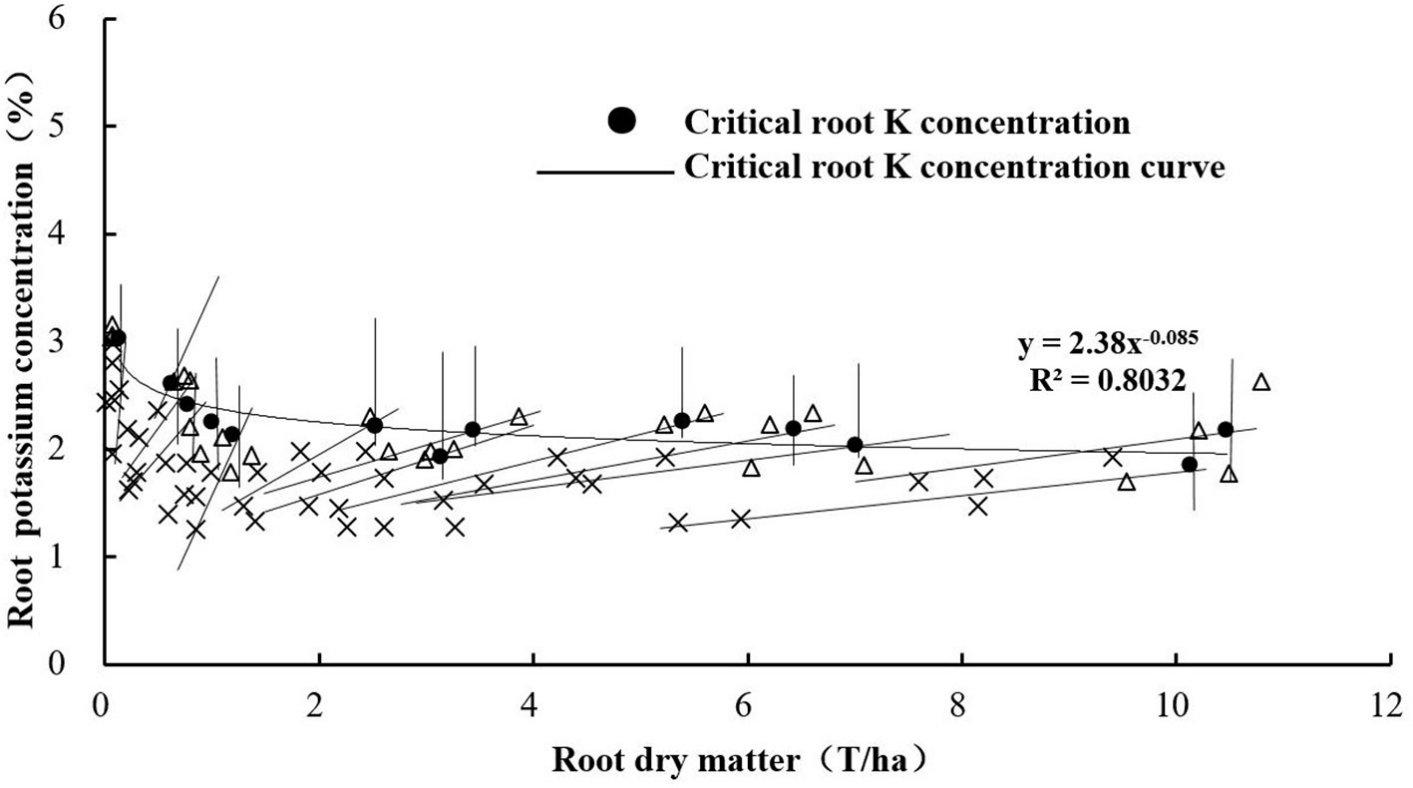
Critical root K concentration curve based on RDM.

The critical root K concentration model based on RDM was also constructed according to the method described in the “Model construction” section. There was a decreasing trend of K_root_ with increasing RDM of sweet potato (Figure 5). The regression trend line could be fitted by the following power equation:


(6) $$\begin{array}{l} {\text{K}}_{\text{root}}=2.38\text{ RD}{\text{M}}^{-0.085} \end{array}$$

### Validation of Critical K Concentration Curves

**Figure 6:**
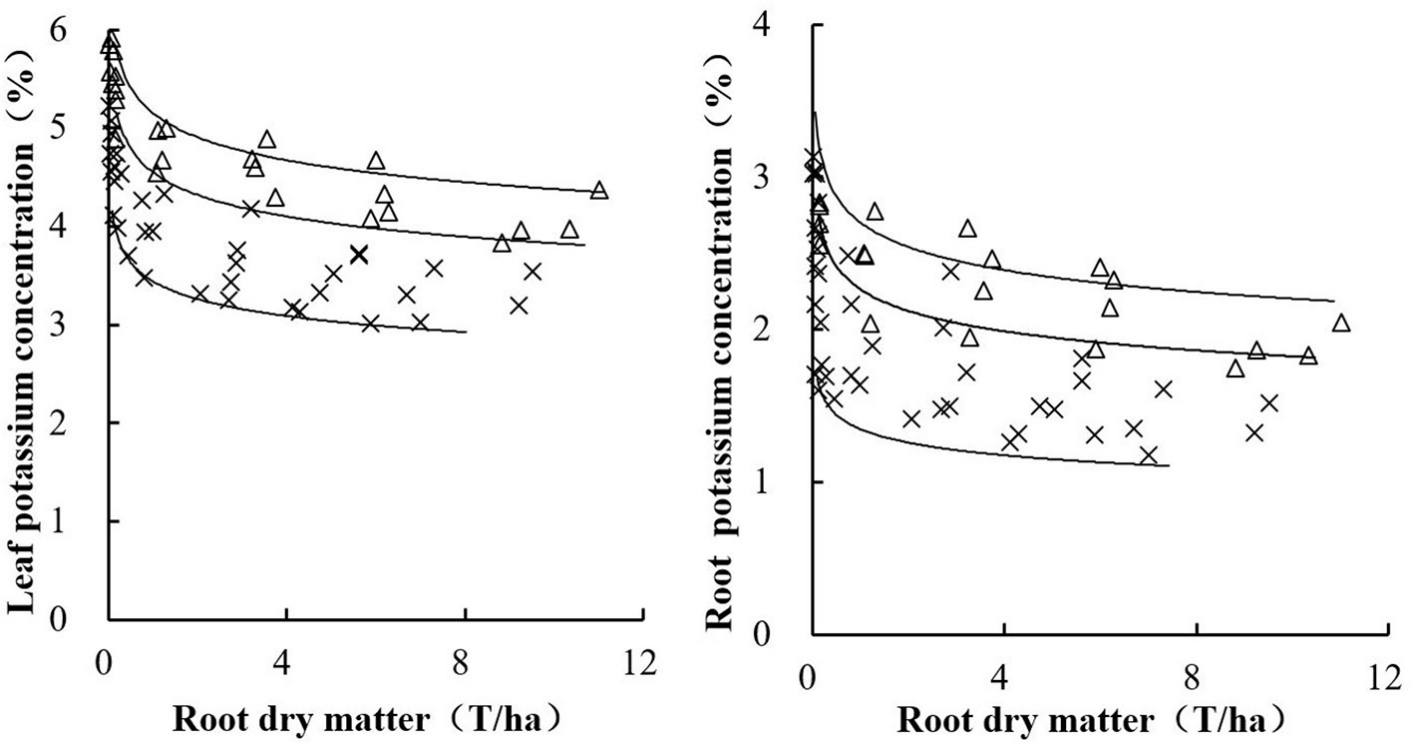
Validation of leaf (left) and root (right) K concentration curves using the independent data set conducted during 2007.

The independent data from an experiment conducted during the 2017 growing season were used for the comprehensive validation of the critical K concentration curves. Both curves identified the situations of K-limiting and non-K-limiting treatments well. The data points from the K-limiting treatments were near or below the curve, while those data points from the non-K-limiting treatments were near or above the curve (Figure 6). Cultivar, growth stage, season, or site did not significantly affect this curve.

The data from the K_4_ and K_0_ treatments in 2018 and 2019 were used to determine the maximum leaf K (K_max1_) and minimum leaf K (K_min1_) curves based on leaf dry matter (LDM). The data from the K_4_ treatments in 2018 and 2019 were used to construct the maximum leaf K (K_max2_) and minimum leaf K (K_min2_) curves based on RDM. The following equations describe the maximum and minimum leaf K concentration curves based on RDM:


(7) $$\begin{array}{lll} {\text{K}}_{\text{max}1} & = & 5.15\text{ RD}{\text{M}}^{-0.07} \end{array}$$

(8) $$\begin{array}{lll} {\text{K}}_{\text{min}1} & = & 3.44\text{ RD}{\text{M}}^{-0.078}. \end{array}$$

The following equations describe the maximum and minimum root K concentration curves based on RDM:


(9) $$\begin{array}{lll} {\text{K}}_{\text{max}2} & = & 2.7\text{ RD}{\text{M}}^{-0.089} \end{array}$$

(10) $$\begin{array}{lll} {\text{K}}_{\text{min}2} & = & 1.35\text{ RD}{\text{M}}^{-0.099}. \end{array}$$

### Differences in the KNI According to K Application Rate

**Figure 7:**
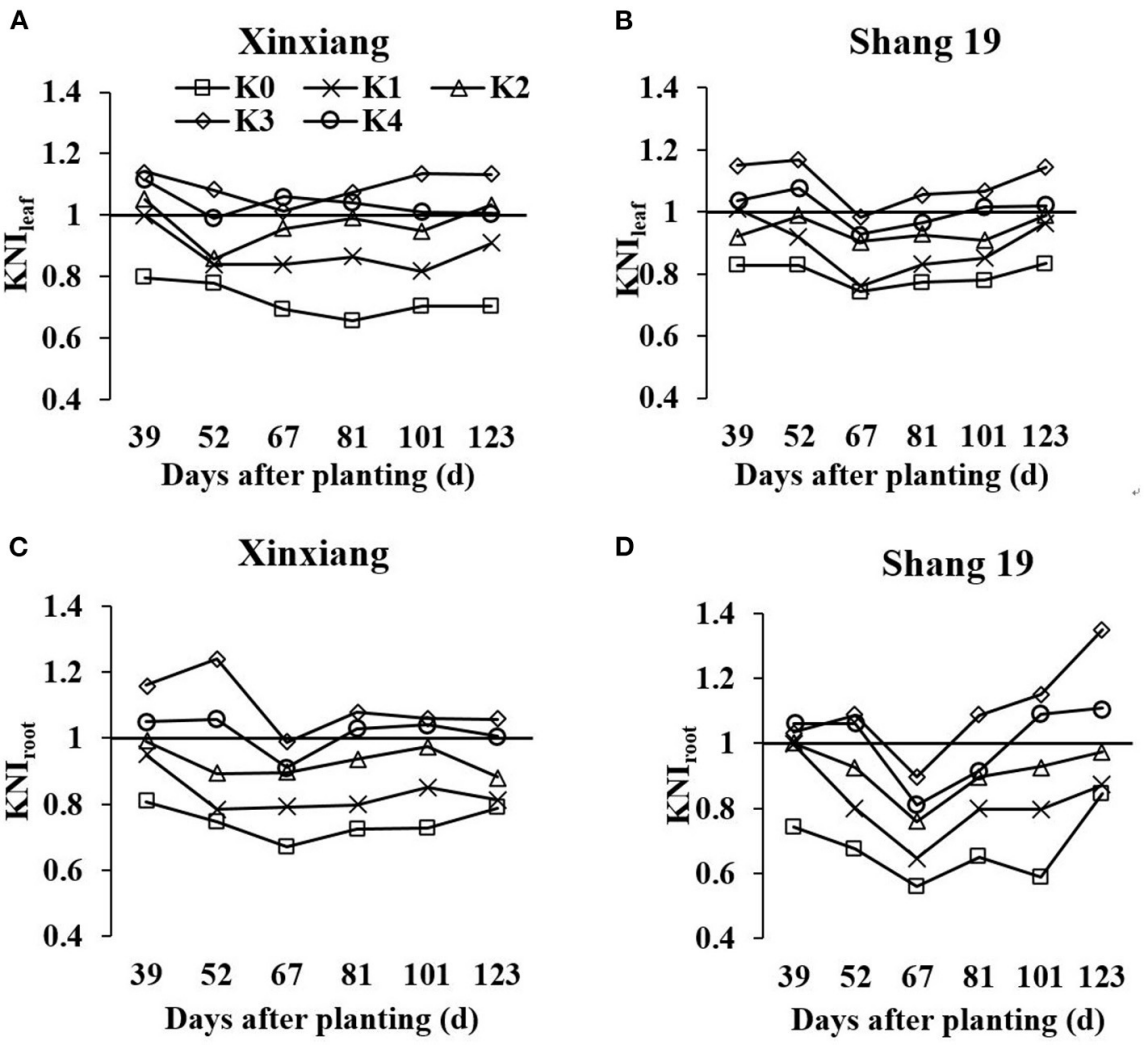
Changes in leaf K nutrition index (KNI) based on leaf dry matter (LDM) and RDM (KNI_leaf_ and KNI_root_, respectively) for sweet potato grown under different K fertilization levels. **(A)** Xinxiang KNI_leaf_, **(B)** Shang 19 KNI_leaf_, **(C)** Xinxiang KNI_root_, and **(D)** Shang 19 KNI_root_.

**Figure 8:**
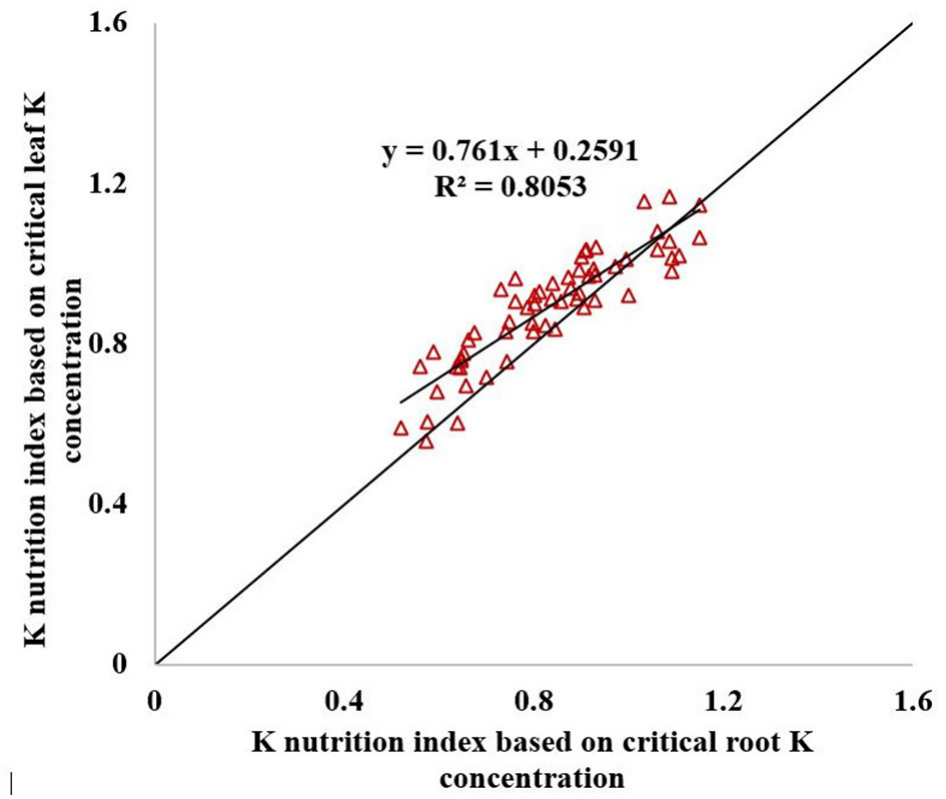
Relationship between KNI_leaf_ and KNI_root_.

The KNI can be used to identify the situations of K-limiting and non-K-limiting treatments. The KNI increased with the increasing K application rate, with the KNI_leaf_ for Shang 19 ranging from 0.76 to 1.17 in 2018, and the KNI_root_ ranging from 0.56 to 1.35 (Figure 7). The KNI_leaf_ for Xinxiang ranged from 0.56 to 1.04 in 2018, whereas the KNI_root_ ranged from 0.52 to 1.09. The KNI_leaf_ values were <1 for the K_0_ and K_1_ treatments, whereas the KNI_leaf_ values were >1 for the K_3_ and K_4_ treatments. The KNI_root_ values were <1 for the K_0_ and K_1_ treatments, whereas the KNI_root_ values were >1 for the K_3_ and K_4_. The KNI values for the K_2_ treatment in 2018 were close to 1, indicating optimal K levels. The minimum KNI value among the K-limiting treatments occurred at the vegetative growth stage. The KNI_root_ values were <1 for the K_2_ treatment during the vegetative growth stage, whereas the KNI_root_ values were >1 during the root expansion stage. The KNI exhibited a decreasing trend at the early stage and then an increasing trend at the latter stage. There was a significant relationship between KNI_leaf_ and KNI_root_ (*R*^2^ = 0.81, Figure 8). In terms of K-deficiency, KNI_leaf_ slightly overestimated the required K nutrition.

### The R/S Ratio

**Figure 9:**
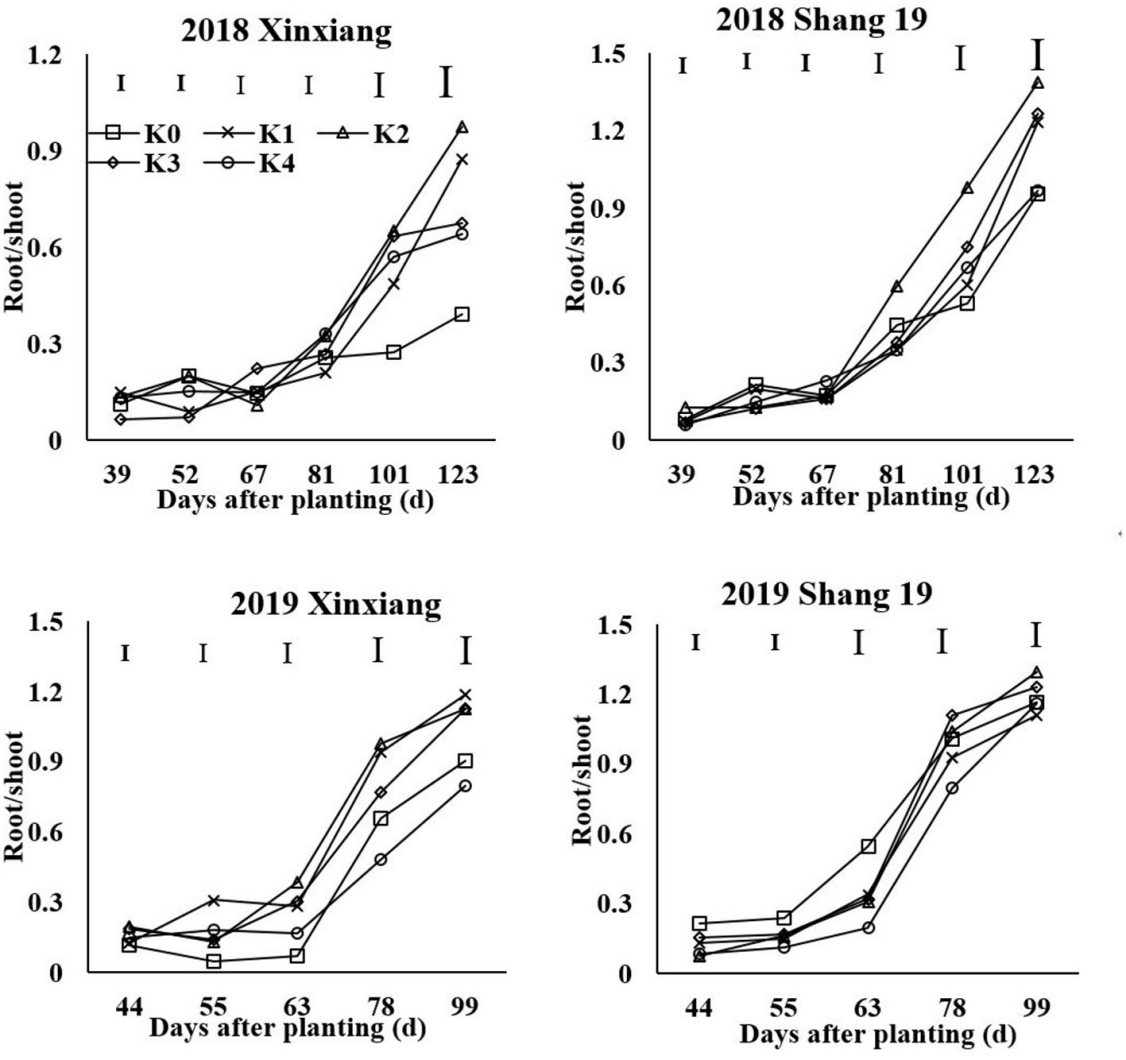
Changes in root/shoot (R/S) ratio with time under different K fertilization levels.

The R/S ratio reflects the relationship in dry matter distribution between the shoots and the roots. The R/S ratio increased gradually during the growth period (Figure 9). With the increase of K application rate, the R/S ratio of sweet potato first increased and then decreased. The R/S ratio of sweet potato in the K_2_ and K_3_ treatments was significantly higher than that in the other treatments. In most cases, the R/S ratio of sweet potato in the K_0_ and K_4_ treatments was lower than that in the other treatments.

**Figure 10:**
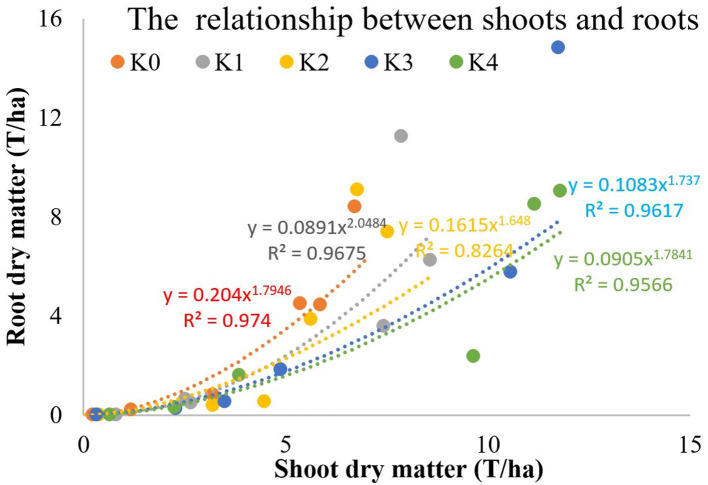
Relationship between shoots and roots under different K fertilization levels.

The allometric growth of root and shoot can be expressed as the following equation: R = a × S^b^ (Figure 10). Low K application inhibited the growth of the storage roots and the growth of the upper plant parts. With the increase of K application rate, the effect of K on the growth rate of the shoot was significantly higher than that of the root. The proportion of dry matter distribution in aboveground was increased by excessive K application.

## Discussion

### Critical K Concentration

Critical nutrient concentration refers to the concentration of a specific nutrient within a specific plant part, below which its growth or yield begins to decline. Based on this approach, a single concentration value is assigned to a point where the plant nutritional status shifts from deficient to adequate (Yin and Vyn, 2004). Smith and Henderson (1985) showed the estimated critical leaf concentrations for K-deficiency in perennial ryegrass (*Lolium perenne* L.) grown in sand culture. Yin and Vyn (2004) showed that the critical leaf K concentration at the initial flowering stage of the development of soybean was 24.3 g kg^−1^. Ramakrishna et al. (2009) developed a diagnostic system by linking the leaf K concentrations to the yield of sweet potato tubers based on empirical statistical methods. In this study, we developed a critical leaf K concentration model to diagnose the K nutrition status of sweet potato. The critical leaf K concentration curve equation was K_leaf_ = 4.55 RDM^−0.075^, whereas the critical root K concentration curve equation was K_root_ = 2.38 RDM^−0.085^. The a_1_ parameter is larger than a_2_, but there was no significant difference between the parameters b_1_ and b_2_. This is because the changes in K concentration in the leaves were consistent with the changes in K concentration in the roots.

Unlike the N dilution phenomenon, K does not enter into a structural pool of the cells but locates in the metabolic pool associated with the enzymatic reactions of respiration, photosynthesis, and translocation of assimilates. Gómez et al. (2018) proved the existence of a “dilution phenomenon” for K in potato and constructed critical shoot dilution curves for K in potato (Suprema: K_c_ = 6.58 W^−0.135^). Adiele et al. (2021) constructed the critical plant and shoot K dilution curves in cassava (the whole plant: K_c_ = 4.2DM^−0.69^; shoot: K_c_ = 4.3DM^−0.54^) and proved that the dilution of K concentration in leaves was less than that in roots, which is consistent with our study. Generally, the K dilution of sweet potato is less than that of potato and cassava. Lv et al. (2020) constructed the following critical N concentration dilution curve equation for roots: N_r_ = 0.57 RDM^−0.425^. The a-coefficient (0.57) for N is much lower than that (2.38) for K, because the concentration of root N stored is much lower than that of K. The b-coefficient (0.425) for N is much higher than that (0.085) for K. This is because K is less diluted than N. Thus, as the plant develops, the roots accumulate an increasing proportion of structural tissues that contain less K.

### The KNI

Soil and tissue analyses are usually used to identify K-deficiency and to predict the K-fertilizer requirements of crops (Shen et al., 1996). However, the recommended amount of K-fertilizer based on the soil-testing results may not be suitable for sweet potato production. Consequently, the plant K content may function as an important alternative index for K-fertilizer management. Walworth and Muniz (1993) classified K contents in potato petiole into three categories, which served to estimate the accurate amount of K-fertilizer for potato plants. Westermann and Tindall (2000) proposed the K diagnostic criteria by comparing petiole K content with the K balance. In this study, the KNI was proposed to diagnose K-deficiency in plants. The KNI is based on the critical K concentration, which has reasonable biological significance.

The KNI highlighted the relationship between actual K concentration and critical K concentration. If the actual K concentration was lower than the critical K concentration, then the sweet potato growth was restricted; the critical K concentration was the most optimal K concentration for maximizing the sweet potato growth and avoiding fertilizer waste. The KNI_leaf_ and KNI_root_ exhibited the same trend during the entire growth period of sweet potato. The correlation coefficient between KNI_leaf_ and KNI_root_ was >0.897. Therefore, KNI_leaf_ can be used to diagnose the K nutritional status instead of KNI_root_.

The KNI exhibited a decreasing trend in 30–60 days after transplanting and then an increasing trend at the latter stages. A large amount of K absorbed was mainly concentrated 30–60 days after transplanting during the tuber formation period. At this stage, the K demand accounted for 54% of the whole growth period (Ning et al., 2011). However, the shoot is the center of growth at this stage (Lv et al., 2020), whereas the root system is small. Therefore, the K absorption capacity is weak, and the KNI decreases at this stage. With the gradual transfer of the growth center to the underground part, the K nutrition absorption capacity of the root system increases, and the KNI increases. The KNI exhibited a significantly increasing trend in 100 days after transplanting. This was because more K was transported from the shoot to the root during the latter stage (Ning et al., 2011). The KNI increased significantly at the latter stage due to K uptake by the root system and transport from the shoot to the root.

**Figure 11:**
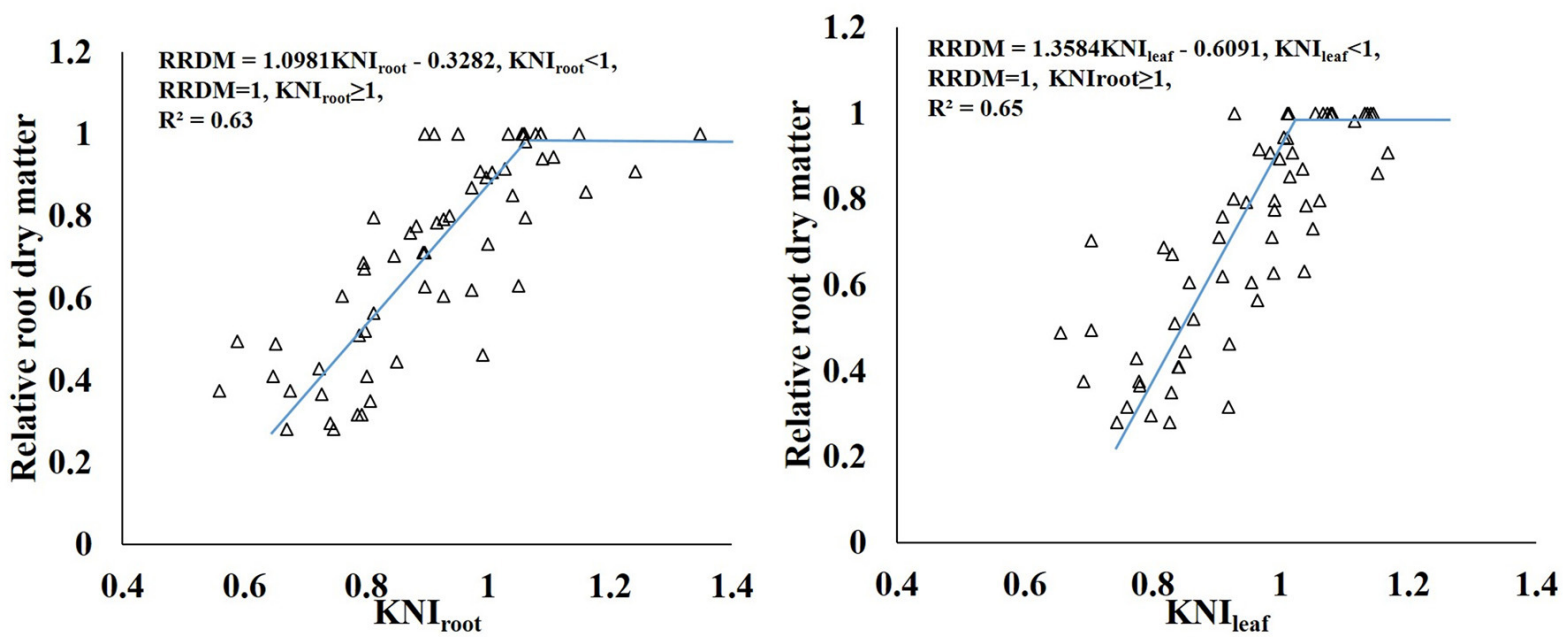
Relationship between relative RDM and the KNI of sweet potato grown under different K rates.

There was a liner-plateau function relationship between relative RDM and KNI. KNI_root_ and KNI_leaf_ explained about 63 and 65% of the variation for the relative RDM, respectively (Figure 11). KNI_leaf_ has better advantages to combine with remote sensing than KNI_root_ for monitoring the yield of sweet potato quickly. Adiele et al. (2021) showed that there was a linear relationship between relative biomass yield and KNI in cassava, and KNI explained 65–66% of the variation for the relative biomass for all treatments. Gómez et al. (2018) developed a quadratic polynomial model between N nutrition index (NNI) and potato yield, and NNI explained 73% of the variability in relative yield. Many other studies also constructed relationships between nutrition index and relative yield (He et al., 2017; Zhao et al., 2018). Therefore, the nutrition index can be used as a diagnostic tool to explain variations in yield caused by differences in crop nutrition status.

### The R/S Ratio

The critical K concentration can reflect the dry matter distribution between shoot and root in sweet potato. K-deficiency first inhibits storage root growth, whereas K excess also inhibits storage root growth to a certain extent (Zhang et al., 2020). The dry matter proportion in roots increased initially and then decreased with the increasing K application rate. The R/S ratio is an important index that reflects the sink-source relationship of sweet potato. The R/S ratio in sweet potato was greater in the K_2_ treatment than in the other treatments. K influenced the storage root yield by an increase in the proportion of dry matter in the storage root and in the storage root numbers per plant. The main reason for the increase in yield following K application was due to the increasing trend of the R/S value, which reflected a larger amount of photosynthate translocation to the storage roots, resulting in their increased size (George et al., 2002; Lu et al., 2003). By contrast, excessive K promoted the growth of the stem slightly (George et al., 2002) and decreased the R/S ratio in sweet potato.

Potassium is the “lubricant” for carbohydrate transfer and distribution between the “sink-source” organs of crops. Proper K supply can regulate the R/S value of sweet potato, promote the transfer of photosynthetic products to root tubers (Ning et al., 2012), and can alleviate any source-sink imbalance. Sweet potato yield depends on both source strength and sink strength. The R/S ratio is an important index that reflects the sink-source relationship of sweet potato. K could significantly increase sucrose phosphate synthase (SPS) activity, sucrose synthase (SS) activity, insoluble acid invertase (IAI), and ADP-glucose pyrophosphorylase (ADPGPPase) activity. The appropriate amount of K could increase leaf SPS activity, chlorophyll content, and improved net photosynthesis (PN), which was beneficial to increase the supply of photosynthates from the source (Ma and Shi, 2011; Wang et al., 2014). Meanwhile, the appropriate amount of K could improve root SS, IAI, and ADPGPPase activity, which promoted the unloading ability of photosynthates to the sink (Liu et al., 2013; Wang et al., 2016). Insufficient leaf K levels under K_0_ treatment lead to decreased photosynthesis. Low K seriously limited the strength of source and sink but have a minimal effect on the R/S ratio (Ning et al., 2013). The R/S ratio under K_2_ treatment reached the maximum. Sweet potato under K_2_ treatment not only supplied more photosynthates but also had higher sink strength of storage roots, which lead to higher photosynthate distribution in storage roots. Compared with the K_2_ treatment, sweet potato under K_4_ treatment has a higher yield but a lower R/S ratio. This was because that high amount of K improved the N accumulation and lead to vigorous aboveground growth. Wang et al. (2016) showed that the total N accumulation under high K treatment (300 mg/kg) increased by 92.1% compared with control in the tuber formation period. Therefore, the excessive K-fertilizer leads to aboveground parts grow excessively and reduce the R/S ratio by greatly increasing the N content in the aboveground parts of sweet potato. In addition, the upper plant parts exhibited a greater range of increased K than the underground parts with increased K concentration (Qi et al., 2016). With increased K concentration, the stems exhibited a greater range of increased K than did the leaves and roots. The excessive K leads to vigorous aboveground growth and consumes most of the photosynthetic products (Li, 2016). Therefore, proper K application helps to regulate the source-sink relationship (Shi et al., 2002; Wang et al., 2017b). The excessive K application decreases the R/S ratio in sweet potato.

### The Advantages of Leaf K Diagnosis

Due to the poor relationship between the soil exchangeable K content and the storage root yield of sweet potato, the recommended amount of K-fertilizer based on the soil-testing results may not be suitable for sweet potato production. The K content in leaves may be an important indicator for K-fertilizer management. However, the traditional test method for determining K content in leaves is not only complex but also time-consuming, which cannot effectively estimate the appropriate amount of K-fertilizer. Therefore, there is a need for a fast and accurate method for the real-time management of K-fertilizer treatment.

Critical leaf nutrient concentrations have often been used to diagnose the causes of crop malnutrition (Westermann and Tindall, 2000; Shi et al., 2018). However, these diagnostic criteria are not available for the mature storage root of sweet potato. Ramakrishna et al. (2009) established the statistical relationship between leaf nutrition and high-yield sweet potato. However, the statistical relationship is not a mechanistic model and cannot show the dynamic changes of K nutrition status. The critical leaf K concentration based on RDM developed in this study belongs to the mechanism model and can show the dynamic changes of K nutrition status at all growth stages of sweet potato. The constructed model in this study provides a reliable means of connecting leaf nutrient concentrations to the storage root yield of sweet potato and is a diagnostic tool that can be used to predict nutrient insufficiencies in sweet potato crops. Therefore, the diagnosis of root K nutritional status using leaf nutrient analysis is recommended.

## Conclusion

A new critical leaf K curves were developed based on the maximum RDM to assess the K nutritional status in sweet potato in China. The critical leaf K curve for sweet potato, based on the maximum RDM, is described by the equation ${\text{K}}_{\text{leaf}}=4.55\times {\text{RDM}}_{\max}^{-0.075}$. The K_leaf_ can diagnose the K status of the storage roots. The curve can effectively identify the situations of K-limiting and non-K-limiting treatments under different conditions. The KNI_leaf_ reflected the relationship between actual K concentration and critical K concentration and can be used to diagnose K-deficiency in plants. Our results show that the critical leaf K concentration curve can provide a reliable means of linking leaf nutrient concentrations to the storage root yield of sweet potato; thus, it is recommended as a reliable indicator for diagnosing the K status in sweet potato.

## Data Availability Statement

The original contributions presented in the study are included in the article, further inquiries can be directed to the corresponding author/s.

## Author Contributions

ZL contributed to designing the experiment, writing, reviewing, and editing. GL was involved in oversight and leadership responsibility for the research activity. All authors contributed to the article and approved the submitted version.

## Funding

This study was supported by the Natural Science Foundation of China (32071897 and 31701322), the China Agriculture Research System of MOF and MARA, and the Key Research and Development Program of Zhejiang Province (2021C02057). Taishun County Science and Technology Planning Project (2021TSXM0049).

## Conflict of Interest

The authors declare that the research was conducted in the absence of any commercial or financial relationships that could be construed as a potential conflict of interest.

## Publisher's Note

All claims expressed in this article are solely those of the authors and do not necessarily represent those of their affiliated organizations, or those of the publisher, the editors and the reviewers. Any product that may be evaluated in this article, or claim that may be made by its manufacturer, is not guaranteed or endorsed by the publisher.
